# Reply to “Triclocarban and Health: the Jury Is Still Out”

**DOI:** 10.1128/mSphere.00255-16

**Published:** 2016-11-02

**Authors:** Lauren Pischel, Angela C. Poole, Catherine Ley, Gina Suh, Julia K. Goodrich, Thomas D. Haggerty, Ruth E. Ley, Julie Parsonnet

**Affiliations:** a Department of Medicine, Stanford University School of Medicine, Stanford, California, USA; b Department of Molecular Biology and Genetics, Cornell University, Ithaca, New York, USA; c Department of Health Research and Policy, Stanford University School of Medicine, Stanford, California, USA; d Department of Microbiology, Cornell University, Ithaca, New York, USA; Arizona State University

## REPLY

We agree with Kennedy et al. (1) that “TCS” was used as an acronym that encompassed both triclosan and triclocarban. Since we provided subjects with health care products containing both substances, ideally we would have measured both as well. However, triclocarban was provided only in hand soaps, and previous studies indicate that little is absorbed through the skin (2). Given low systemic levels, it seemed implausible that triclocarban would have a major effect on either the gut or oral microbiome. For these reasons, we do not believe triclocarban levels—which likely would have been negligible—would have altered our paper’s conclusions. We do plan to assess the skin microbiome, however, where triclocarban’s effects are most likely to be manifest. We look forward to reporting those results in the future.
